# Effects of mannan oligosaccharide supplementation on growth, some immune responses and gut lactic acid bacteria of common carp *(Cyprinus Carpio)* fingerlings

**Published:** 2015-09-15

**Authors:** Parvin Momeni-Moghaddam, Saeed Keyvanshokooh, Saeed Ziaei-Nejad, Amir Parviz Salati, Hossein Pasha-Zanoosi

**Affiliations:** 1 *Department of Fisheries, Faculty of Marine Natural Resources, Khorramshahr University of Marine Science and Technology, Khorramshahr, Iran;*; 2 *Department of Fisheries, Faculty of Natural Resources, Behbahan Khatam Alanbia University of Technology, Behbahan, Iran; *; 3 *Department of Physical Oceanography, Faculty of Marine Sciences, Khorramshahr University of Marine Science and Technology, Khorramshahr, Iran.*

**Keywords:** *Cyprinus carpio*, Growth, Innate immunity, Intestinal microbiota, Mannan oligosaccharide

## Abstract

This study was conducted to determine the effects of mannan oligosaccharide (MOS) on growth, some immune responses and gut lactic acid bacteria of common carp. Four experimental diets containing 0%, 0.05%, 0.10% and 0.20% MOS were prepared. Each diet was randomly allocated to triplicate groups of fish with initial average weight of approximately 14 g. After eight weeks, survival rate was high in all treatments with no significant difference (*p* > 0.05). Growth performance including final weight, weight gain (WG) and specific growth rate (SGR) did not differ among the treatments. Feed conversion ratio (FCR) was better when the fish were fed 0.05 to 0.20% MOS diets. The alternative complement activity, lysozyme activity and serum total Ig were found to be significantly (*p *< 0.05) greater in fish fed 0.20% MOS diets. Although the total intestinal bacterial counts were not affected by dietary treatment (*p* > 0.05), the lactic acid bacteria levels were significantly elevated in fish fed MOS diets (*p *< 0.05). These results indicated that oral administration of MOS at 0.20% elevated the immune response, improved FCR and modulated intestinal microbiota of common carp.

## Introduction

Antibiotics, for the past decades, have been included at the sub-therapeutic concentrations in the aqua-feeds due to their potential effects on survival, feed utilization and weight gain.^1^ However, with the recent ban on the use of antibiotic growth promoters as additives in fish feed, alternative immunonutriens to enhance production and health status is a research topic of increasing interest. Prebiotics which are defined as non-digestible food ingredients beneficially affect the host by stimulating growth and/or activity of a limited number of beneficial bacteria in the gastrointestinal tract and have proved to be effective at enhancing health and growth performance of terrestrial and aquatic animals.^2^

Among the most common prebiotics, mannan oligosaccharide (MOS) has been recently receiving heightening application in aquaculture.^1^ The MOS are complex carbohydrates derived from yeast cell walls. These compounds contain mannose as the primary carbohydrate element.^3^ This prebiotic has been demonstrated to have a variety of beneficial effects on livestock ranging from growth enhancing to immunostimulation in many species.^4^ The growth promotion^5^^-^^7^ and immuno-stimulatory benefits^8^^,^^9^ of dietary MOS have also been studied in fish but often with contrasting findings. 

The culture of warm-water species is an important aquaculture industry in Iran. Warm-water fish farming in Iran is based on Chinese carps including common carp (*Cyprinus carpio*). Annual production of farmed carps in Iran was 100,430 tons in 2009.^10^ The present study was carried out to investigate the effects of dietary MOS supplementation on the growth performance and immunological parameters of common carp.

## Materials and Methods

**Fish and experimental conditions. **Three hundred common carp fingerlings were obtained from a commercial carp farm in Shoushtar, Iran. The fish were transported to the laboratory, acclimated to laboratory conditions and fed the basal experimental diet without dietary MOS for one week. At the end of the acclimation period, fish with an average weight of approximately 14 g were randomly selected and stocked in twelve 300-L tanks (triplicate groups per dietary treatment) at a density of 20 fish per tank. The mean water quality parameters were recorded as follows: temperature 27 ^°^C, dissolved oxygen 8.2 mg L^-1^ and pH 7.9.

**Feed and feeding. **Basal diet formulation and proximate composition analysis are shown in Table 1. The experimental diet was formulated to have 0% (basal), 0.05%, 0.10% and 0.20% of mannan oligosaccaride (MOS; Alltech Inc., Nicholasville, USA). Dietary ingredients were thoroughly mixed in a mixer, made into pellets and air-dried at room temperature. The pellets were broken into small pieces, packed and stored in a freezer until used.

Fish were fed by hand to apparent satiation two times per day with one of the four experimental diets over eight weeks. 

**Table 1.** Ingredients composition of test diets fed by common carp

**Ingredient **	**Control diet**	**0.05%**	**0.10%**	**0.20%**
***(% dry weight)*** **Fish meal **	37.50	37.50	37.50	37.50
**Wheat flour**	10.00	10.00	10.00	10.00
**Wheat bran**	14.00	14.00	14.00	14.00
**Soybean meal**	16.50	16.50	16.50	16.50
**Starch**	15.00	15.00	15.00	15.00
**Soybean oil **	6.00	6.00	6.00	6.00
**Mineral premix** a	0.50	0.50	0.50	0.50
**Vitamin premix** b	0.50	0.50	0.50	0.50
**Prebiotic MOS**	0.00	0.50	1.00	2.00
***Analyzed Proximate composition (% dry weight)***				
**Crude protein **	32.30	32.30	32.30	32.30
**Crude lipid**	13.30	13.30	13.30	13.30
**Fiber**	4.47	4.49	4.50	4.50
**Ash**	14.10	14.10	14.10	14.10
** Energy (Kcal g** ^-1 ^ **diet) **	3.75	3.79	3.79	3.80

a Unit kg^-1^ of mixture: Mineral: Fe, 26 g; Cu, 4.2 g; Co, 480 mg; Se, 2 g; Zn, 12.5 g; Mn, 15.8 g; I, 1 g; choline chloride, 12 g.

b Unit kg^-1^ of mixture: Vitamins: Retinol acetate (A) 160000 IU; Cholecalciferol (D_3_) 400000 IU; DL-α-tocopheryl acetate (E) 40 g; Menadione sodium bisulfite (K_3_) 2 g; L-ascorbic acid (C) 60 g; D-Biotin (H_2_) 0.24 g; Thiamin mononitrate (B_1_) 6 g; Riboflavin (B_2_) 2 g; Niacinamide (B_5_) 40 g; Pyridoxine hydrochloride (B_6_) 4 g; Folic acid (B_9_) 2 g; Cyanocobalamin (B12) 8 g; Inositol 20 g.

**Growth measurements. **All fish in the different experimental groups were weighed at the end of 8-week feeding trial for estimation of growth. Based on recording the weight of each fish, specific growth rate (SGR), percentage of body weight gain (WG) and feed conversion ratio (FCR) were calculated for each group as follows: 

Specific growth rate =100×Ln final weight –Ln initial weightTotal duration of the experiment

$\mathrm{Weight gain}=100\times \frac{\mathrm{Final body weight}–\mathrm{initial body weight}}{\mathrm{Initial body weight}}$

$\mathrm{Feed conversion ratio}=\frac{\mathrm{Feed given}(\mathrm{dry weight})}{\mathrm{Weight gain}(\mathrm{wet gain})}$

In addition, survival rate was calculated at the end of the experiment as: 

$\mathrm{Survival rate}=\frac{N_{f}}{N_{o}}\times 100$

where, *N*_0_ is the initial number of fish and *N*_f_ is the final number of fish.

**Immunological assays.** After eight weeks of feeding, blood samples for serum biochemical analysis were collected from the caudal vein of nine fish per each dietary treatment into Eppendorf tubes (Eppendorf, Hamburg, Germany) without anticoagulant. Blood samples in Eppendorf tubes were allowed to clot for 30 min at room temperature. The tubes were then centrifuged at 3500 *g* for 5 min and the supernatant serum was collected. The serum was kept frozen at – 20 ˚C until analysis of selected biochemical parameters.

**Alternative complement activity (ACH**_50_ **). **The 50% hemolysis was determined using the method of Sunyer and Tort ^11^ with the following modifications as described by Yeh *et al.*^12^ The volume of serum complement producing ACH_50_ was determined, and the number of ACH_50_ unit mL^-1^ was calculated for the sample. 

**Lysozyme activity. **Lysozyme activity in serum was determined according to the method of Demers and Bayne^13^ based on the lysis of the lysozyme sensitive Gram positive bacterium, *Micrococcus lysodeikticus* (Sigma Chemical Co., St Louis, MO, USA). The dilutions of hen egg white lysozyme (Sigma Chemical Co.) ranging from 0 to 20 µL mL^-1^ in 0.1 M phosphate citrate buffer (Merck, Darmstadt, Germany), pH 5.8, were taken as the standard. This along with the undiluted serum sample (25 µL) was placed into wells of a 96-well plate in triplicate. A volume of 175 µL of *M. lysodeikticus* suspension (75 mg mL^-1^) prepared in the same buffer was then added to each well. After rapid mixing, the change in turbidity was measured every 30 sec for 5 min at 450 nm at approximately 20 ^°^C using a microplate reader (Model Stat Fax-2100; Awareness Technology, Palm City, USA). 

**Total immunoglobulin content. **Plasma total immunoglobulin (Ig) level was determined according to the method described by Puangkaew *et al.*^14^ The assay was based on the measurement of total protein content in plasma prior to and after precipitating the Ig molecules employing a 12.0% solution of polyethylene glycol (Sigma Chemical Co.). The difference in protein content represents the Ig content.

**Intestinal lactic acid bacteria analysis**. The analysis of intestinal microbiota was performed at the end of the feeding trial as described by Hoseinifar *et al.* ^15^ with some modifications. Four fish per tank were randomly sampled from each treatment for microbiological sampling. The fish were killed by physical destruction of the brain, and the skin was then washed in a solution of 0.10% benz-alkonium chloride before opening the ventral surface with sterile scissors. Intestinal tract samples of fish were removed, weighed, and suspended in sterile 0.85% NaCl solution (Merck, Darmstadt, Germany) and homogenized. The suspension, serially diluted to 10^-6^ and 20 µL of the solution was spread in triplicate on to nutrient agar (NA; Acumedia Neogen Co., Lansing, USA). Man Rogosa and Sharpe media (MRS; Quelab, Montreal, Canada) was also used to detect lactic acid bacteria (LAB). All of the plates were incubated at 36 ˚C for three days and colony forming units (CFU) per g were calculated. 

**Statistical analysis. **The data (Mean ± standard error) were analyzed by one way analysis of variance (ANOVA) followed by Tukey’s test to compare the means between individual treatments with SPSS (Version 16; SPSS Inc., Chicago, USA) at *p *< 0.05 level.

## Results

**Survival and growth measurements. **At the end of trial, survival rate was high in all treatments with no significant difference (*p* > 0.05). The growth performance of common carp fingerlings fed diets supplemented with different levels of dietary MOS is presented in Table 2. After eight weeks of feeding, growth performance including final weight, WG and SGR did not differ among the dietary treatments, however, FCR was better when the fish were fed 0.05 to 0.20% MOS supplemented diets. 

**Table 2:** Growth performance of common carp fingerlings fed dietary mannan oligosaccharide (MOS) at the end of 8^th^ weeks of feeding trial. Values are mean ± SE of three replicate groups

**Parameters**	**Control**	**0.05%**	**0.10%**	**0.20%**
**Final weight (g)**	18.11 ± 0.22^a^	17.42 ± 0.34^a^	18.42 ± 0.06^a^	18.01 ± 0.88^a^
**Weight gain (%)**	30.19 ± 1.22^a^	23.02 ± 1.34^a^	34.44 ± 1.06^a^	30.13 ± 1.88^a^
**Specific growth rate**	0.19 ± 0.028^a^	0.15 ± 0.031^a^	0.22 ± .012^a^	0.19 ± 0.039^a^
**Feed conversion ratio**	2.83 ± 0.05^b^	2.40 ± 0.07^a^	2.41 ± 0.07^a^	2.27 ± 0.10^a^
**Survival (%)**	96.66 ± 5.77^a^	93.3 ± 2.88^a^	96.66 ± 2.88^a^	96.66 ± 5.77^a^

ab Mean values with different superscripts in each row are significantly different from each other (*p* < 0.05).

**Fig. 1:**
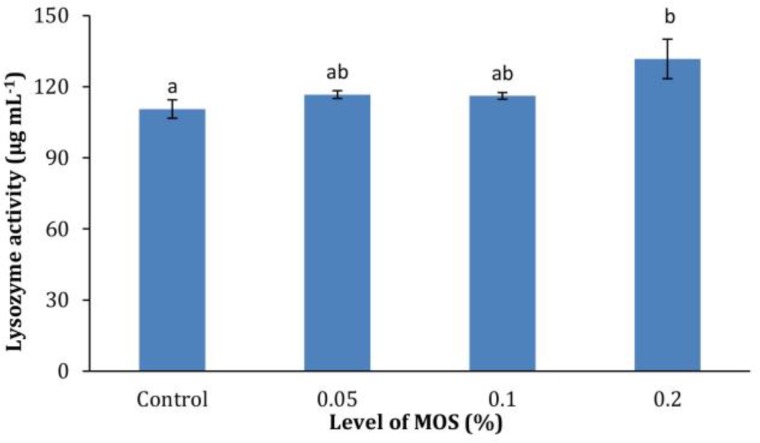
Serum lysozyme activity of common carp fed different levels of dietary mannan oligosaccharide (MOS). Data are presented as mean with standard deviation as error bars.

^ab^ Different letters indicate significant difference (*p* < 0.05).

**Fig. 2:**
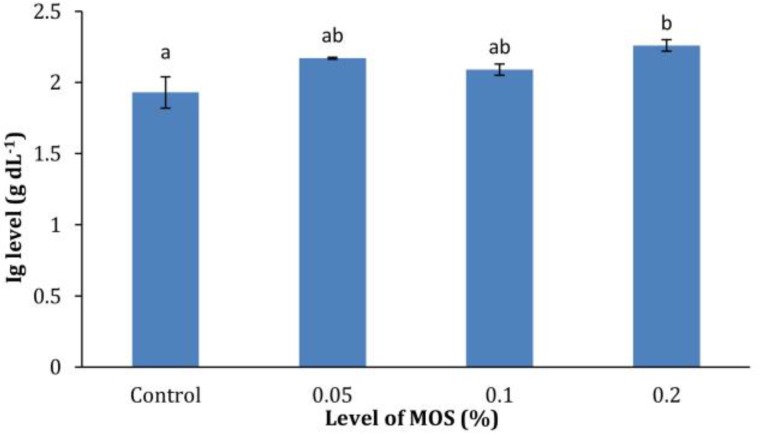
Plasma total immunoglobulin (Ig) levels of common carp fed different levels of dietary mannan oligosaccharide (MOS). Data are presented as mean with standard deviation as error bars.

^ab^ Different letters indicate significant difference (*p* < 0.05).

**Fig. 3:**
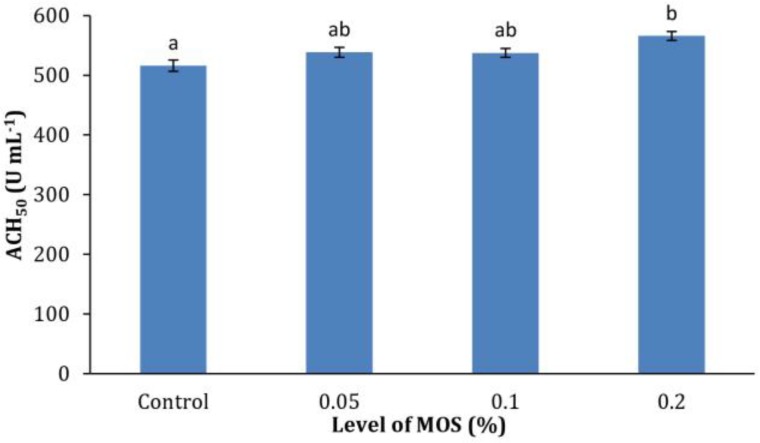
Serum alternative complement activity (ACH_50_) of common carp fed different levels of dietary mannan oligo-saccharide (MOS). Data are presented as mean with standard deviation as error bars.

^ab^ Different letters indicate significant difference (*p* < 0.05).

**Immunological assays.** The effects of the MOS on the humoral innate immune responses of common carp are shown in Figs. 1, 2, and 3. All immune parameters measured (lysozyme activity, Ig and alternative pathway of complement activity) were significantly higher (*p* < 0.05) in 0.2% MOS fed fish compared to the control group. Fish fed 0.20% MOS showed significantly elevated lysozyme activity compared to the control, but the activity was not significantly higher than the 0.05% and 0.10% MOS groups, as was also the case for plasma Ig and ACH_50_ levels.

**Fig. 4:**
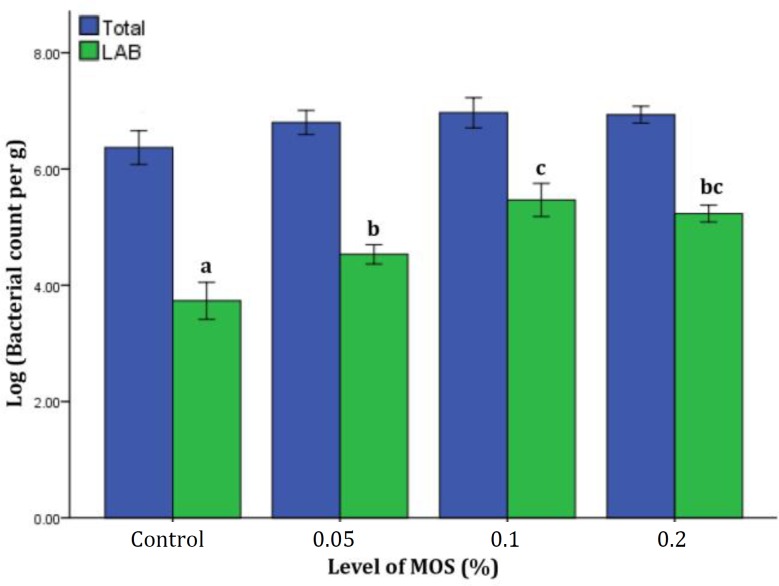
Total culturable bacteria and LAB levels (log CFU per g of intestine) of common carp fed different levels of dietary mannan oligosaccharide (MOS). Data are presented as mean with standard deviation as error bars.

**Intestinal microbiota. **Intestinal microbiota data are shown in Fig. 4. At the end of feeding trial, total bacterial levels were not affected by MOS (*p* > 0.05). Levels of LAB were significantly elevated in MOS fed fish compared to the control group (*p* < 0.05).

There is no significant difference in terms of total culturable bacteria based on different levels of MOS.

^abc^ Different letters indicate significant difference in terms of LAB levels (*p* < 0.05).

## Discussion

This study was conducted in order to investigate the effects of MOS on growth, survival and immune responses of common carp. From previous studies it appears that the effects of MOS on growth performance of aquatic species are contradictory. The results of the current study indicate that dietary MOS supplementation at 0.05, 0.10 and 0.20% had no significant effects on growth performance of common carp fingerlings. Similar to the results of the present study are previous investigations by Mansour *et al.* ^16^ on giant sturgeon (*Huso huso*) fed 0, 2 and 4 g kg^-1^ MOS, Dimitroglou *et al.* ^17^ on gilthead sea bream (*Sparus aurata*) treated with different levels of MOS (0, 2.00 and 4.00%), Pryor *et al.* on Gulf sturgeon (*Acipenser oxyrinchus desotoi*) fed 0 and 3 g kg^-1^ mannan oligo-saccharide,^5^ Genc *et al.* on African catfish (*Clarias gariepinus*) fed diets with 1.00, 2.00 and 3.00 g kg^-1 ^MOS for 80 days,^18^ which all showed no significant differences in growth parameters between fish fed the control and MOS supplemented diets. On the contrary, Staykov *et al.* used dietary MOS to examine the effect on growth performance of rainbow trout and found significantly improved growth performance.^19^ Additionally, Torrecillas *et al.* also demonstrated that administration of two levels of MOS (0.20 and 0.40%) in diet of juvenile European sea bass (*Dicentrachus labrax*) makes 10.00% improvement in growth performance.^20^ Based on current knowledge, the contradictory results may be attributable to the MOS dosage, different duration of oral administration, life stage and/or different fish species.

In commercial aquaculture, the farmed species are vulnerable to ubiquitous opportunistic bacterial pathogens and stimulation of the innate immune system through dietary supplements is of great interest. The alternative pathway of complement activity acts as a powerful non-specific defense mechanism, protecting fish from a wide range of potentially invasive organisms, such as bacteria, fungi, viruses, and parasites.^21^ Lysozyme, being an enzyme with antimicrobial activity, can split peptidoglycan in bacterial cell walls especially of the Gram positive species and can cause lysis of the cells.^22^ As presented in Figs. 1 and 3, fish fed 0.20% MOS for eight weeks showed increased lysozyme activity and ACH_50_. Additionally, increased level of serum total Ig as the major components of the humoral immune system was detected in the serum of fish fed 0.20% MOS (Fig. 2). The results are consistent with findings of previous studies on shellfish ^1^^,^^23^ and fish species. Staykov *et al.* reported higher levels of bactericidal activity, lysozyme, antibody levels and alternative complement activity in rainbow trout fed MOS.^19^ In addition, similar results have also been demonstrated in European sea bass fed 0.40% MOS supplemented diets.^20^ Similarly, other prebiotics such as inulin and fructo-oligosaccharide (FOS) have been demonstrated to improve the innate immune response in other fish species.^15^^,^^24^^,^^25^ The positive effects of prebiotics on humoral immune responses may be attributable to improved growth of health-promoting bacteria such as *Lactobacillus* and *Bifidobacter *spp. that consequently limit potentially pathogenic bacteria.^2^

Our results based on analysis of intestinal microbiota revealed that LAB levels were significantly elevated in fish fed dietary MOS (*p *< 0.05). Prebiotics including MOS have been reported to enhance the growth of beneficial bacteria such as LAB.^2^ Regarding the improvement of innate immune parameters measured in our study, it can be concluded that modulation of the intestinal microbiota towards a potentially more beneficial microbial community (i.e. elevated LAB levels) can be achieved through administration of dietary MOS.

In conclusion, the results of this study show that oral administration of MOS at 0.05-0.20% improves FCR, modulates intestinal microbiota and at 0.20% elevates the humoral immune response of common carp by increasing the alternative complement activity, lysozyme activity and serum total immunoglobin. 
